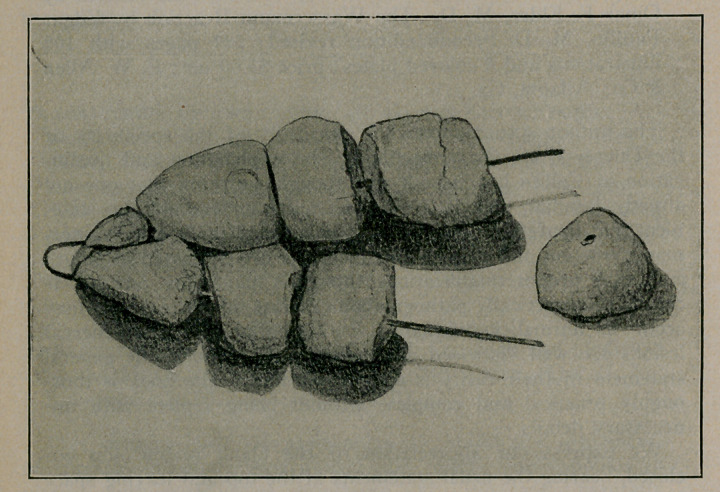# Extraordinary Case of Calculi Formed on a Foreign Body

**Published:** 1914-02

**Authors:** 


### ABSTRACTS

---

Extraordinary Case of Calculi Formed on a Foreign Body. Dr. G. W. Maly reports the following interesting case (*Zeit. für. Gyn., Urol. Amer. Jour. Urology,* June, 1913.) The patient was a girl of 22, who during the past year had been complaining of pains in the abdomen and constantly increasing urinary difficulties. On examination the hymen was found intact. Right behind the symphysis in the anterior vaginal wall one could feel a hard nodular resistance. The catheter at once encountered a hard body. The urine was turbid, ammoniacal. Cystoscopically one could plainly see at once a hairpin with seven stones strung out regularly over both prongs. The size of the stones was that of hazel nuts and they had well formed facets. An eighth stone, which had separated from the hairpin, was lying nearby The fine lumen of the stone, showing that it was also formed on the hairpin, can still be seen. Suprapubic cystotomy was performed, recovery was uneventful, and after 14 days the patient was discharged well.

The illustration reproduces the stones in their arrangement and their natural size. The surface of the stones is yellowish red, somewhat rough but smooth at the facets. The stones were not fixed on the pin but could be turned and moved. The hairpin was introduced for purposes of masturbation. We acknowledge the courtesy of the *Critic and Guide* in furnishing the cut.

## Figures and Tables

**Figure f1:**